# Lumen-Apposing Metal Stent as a Salvage Therapy for Corrosive-Induced Gastric Outlet Obstruction: A Case Report

**DOI:** 10.5152/tjg.2026.25719

**Published:** 2026-08-18

**Authors:** Mucahit Ergul, Oguz Ozturk, Mehmet Rasit Ayte, Kadir Can Acun, Emir Tugrul Keskin, Ali Atay, Yavuz Cagir, Ilhami Yuksel

**Affiliations:** 1 Department of Gastroenterology, Ankara Bilkent City Hospital, Ankara, Türkiye; 2 Department of Gastroenterology, Ankara Sanatorium Training and Research Hospital, Ankara, Türkiye; 3 Department of Gastroenterology, Ankara Yenimahalle Training and Research Hospital, Ankara, Türkiye; 4 Department of Gastroenterology, Ankara Yıldırım Beyazit University School of Medicine, Ankara, Türkiye

## Case Presentation

Caustic-induced gastric outlet obstruction (GOO) results from transmural injury followed by intense submucosal fibrosis and cicatrization.^1^ Endoscopic therapy demonstrates less favorable outcomes because patients require multiple dilation sessions and commonly necessitate surgical intervention.^2^^,^ ^3^ The use of lumen-apposing metal stent (LAMS) for caustic-induced GOO is not an established treatment. The authors present their experience with this technique as rescue therapy extrapolated from its use in related benign indications. This case report was prepared and reported in accordance with the CARE guidelines. Informed consent was obtained.

**Figure 1.**
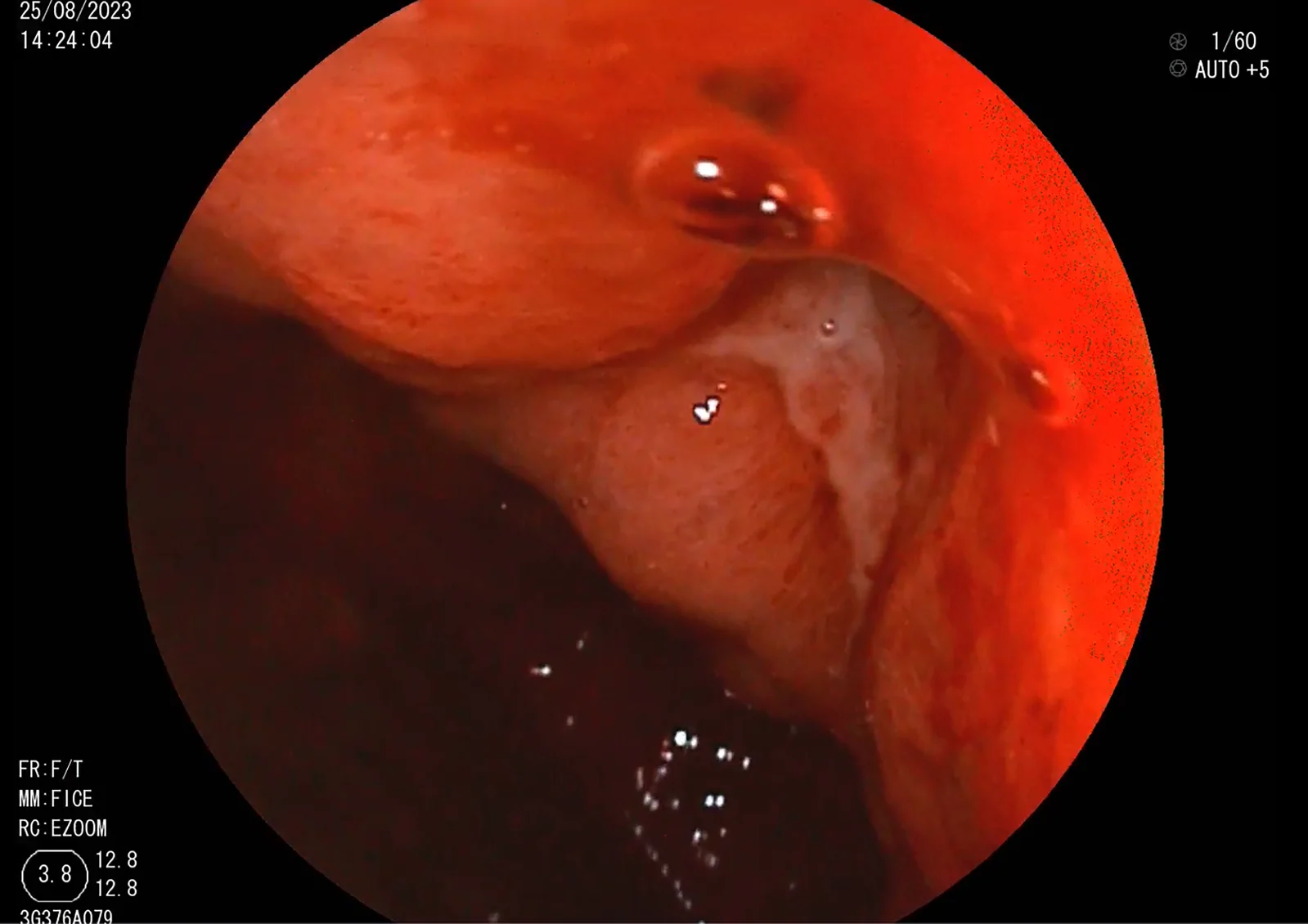
Upper endoscopy revealed a strictured pyloric channel with exudative erosions and edema.

**Figure 2.**
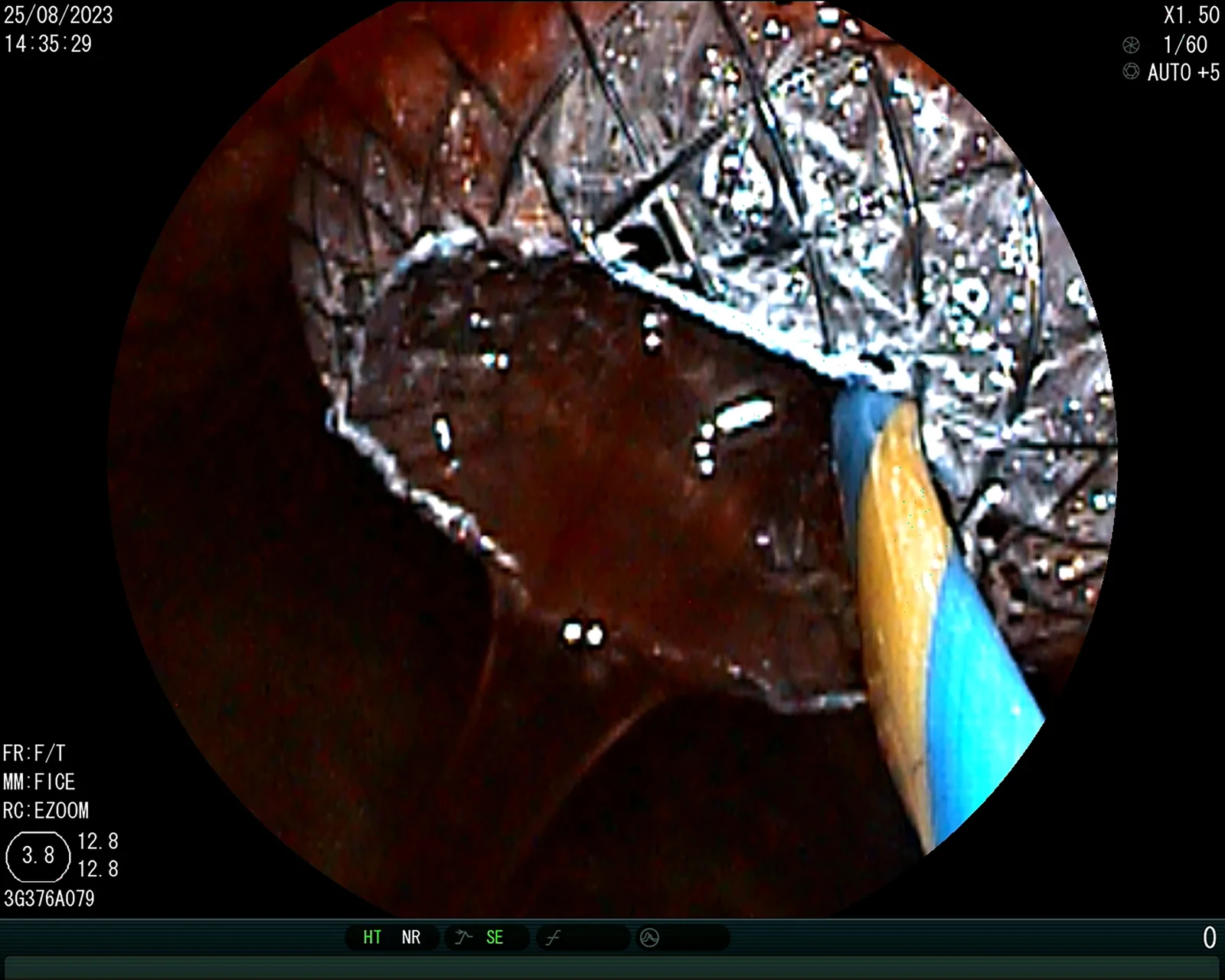
The lumen-apposing metal stent was placed using dual-channel gastroscope under fluoroscopic guidance.

**Figure 3.**
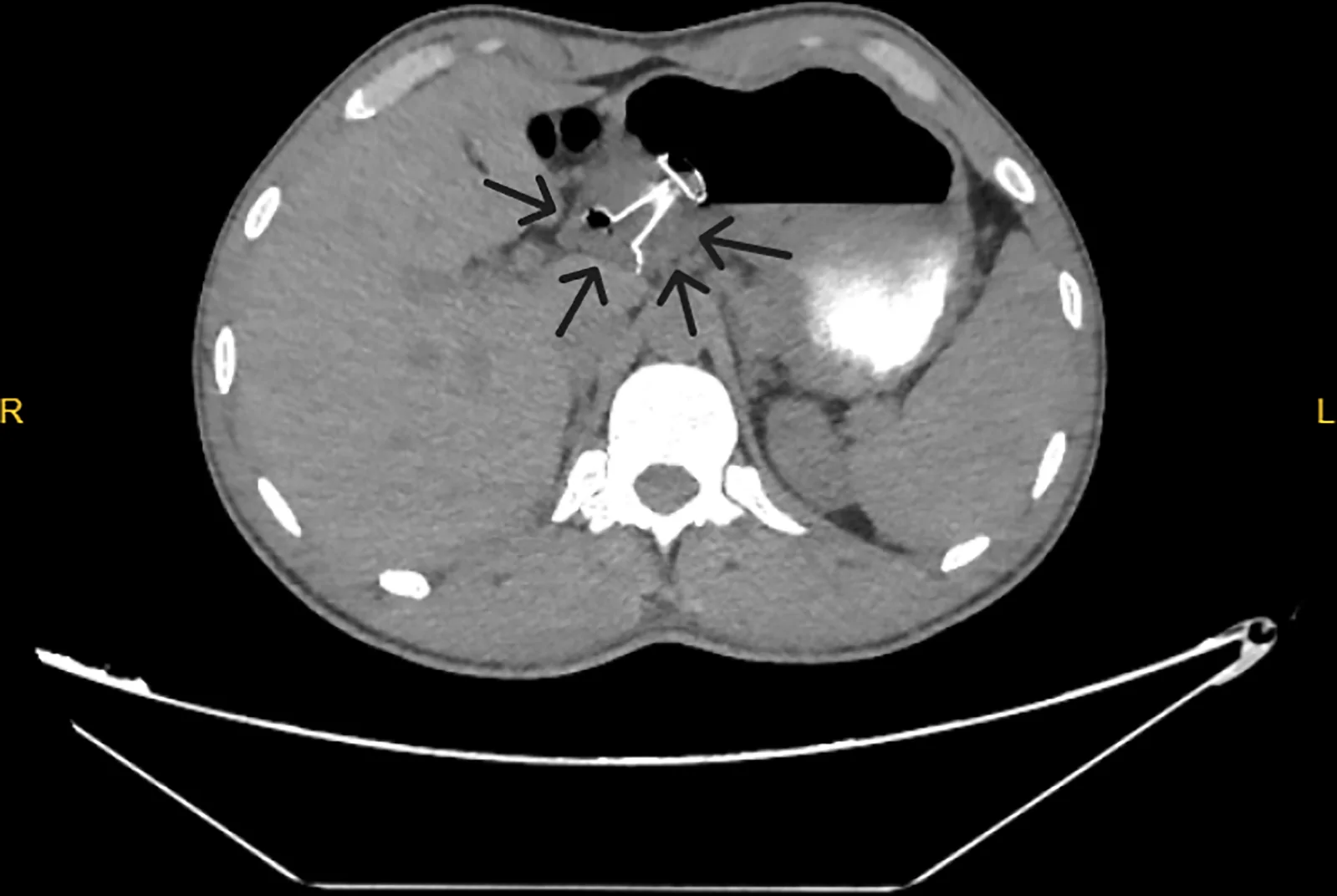
Computed tomography image showing the lumen-apposing metal stent deployed at the pylorus. Arrows indicate the precise location of the stent across the stenotic segment.

A 32-year-old man presented to the emergency department after accidentally ingesting a hydrochloric acid–based liquid (Betonax fix concrete remover), with nausea, vomiting, and abdominal pain. Upper endoscopy revealed a strictured pyloric channel with exudative erosions and edema (Figure 1), consistent with Zargar grade 2a injury; the remaining segments appeared normal. Balloon dilatation was performed 4 weeks after caustic ingestion; however, obstructive symptoms recurred within 24 hours. Therefore, a fully covered self-expanding metallic stent (FCSEMS) (10 × 60 mm, Micro-Tech, Nanjing, China) was placed without an antimigratory technique; however, it migrated into the stomach 3 days later. Consequently, the authors elected to place an LAMS to maintain pyloric channel patency. Fluoroscopy using an opaque insufflated balloon revealed isolated pyloric stenosis. The LAMS (16 × 20 mm, Micro-Tech) was placed using a dual-channel gastroscope (Fujinon EG-530D, Fujifilm, Japan) under fluoroscopic guidance (Figure 2). Computed tomography confirmed the LAMS in the pylorus (Figure 3). The following day, the patient tolerated a soft diet without symptoms. At the follow-up endoscopy, LAMS was fully expanded and remained securely positioned across the pylorus.

**Figure 4.**
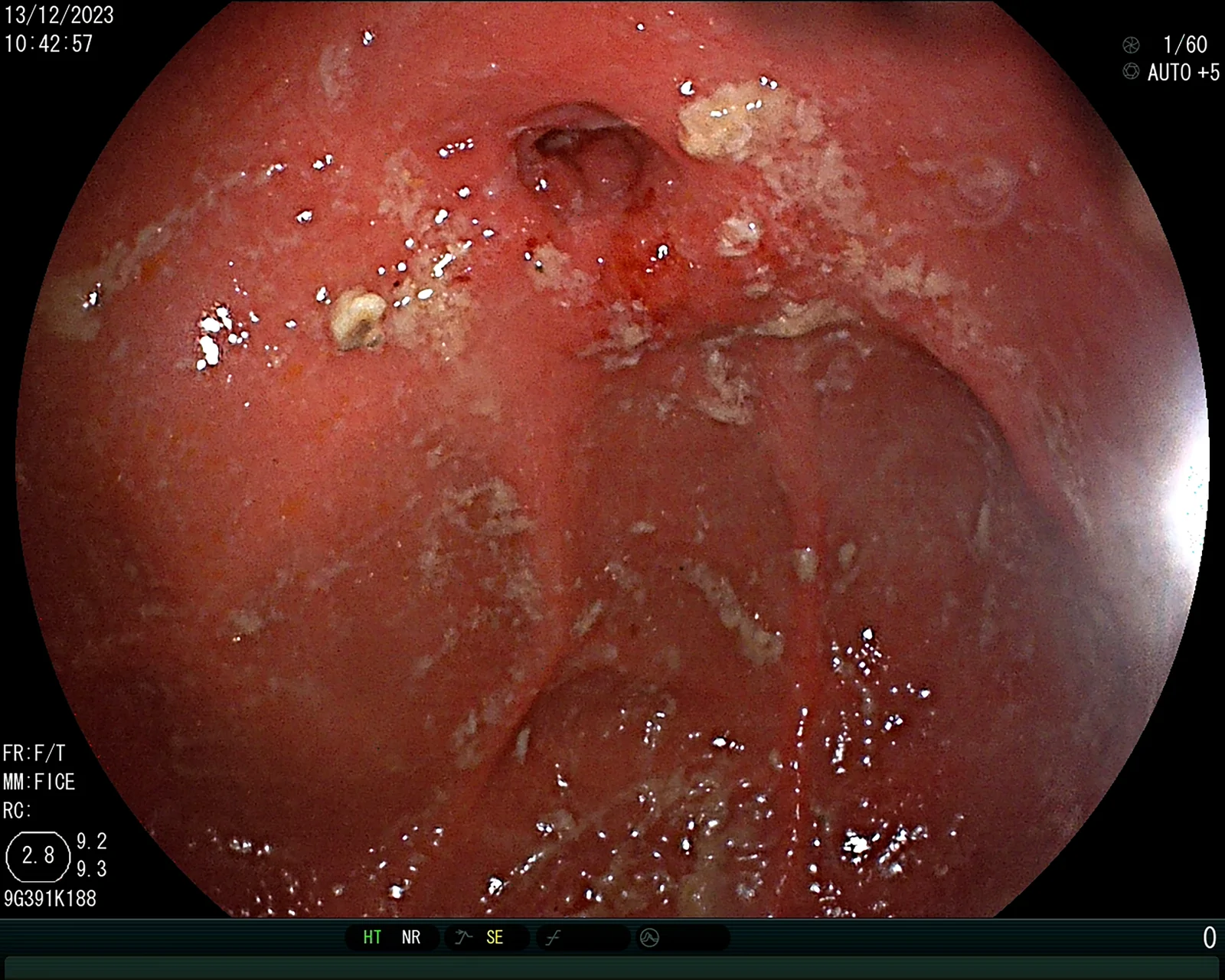
One month later, the lumen-apposing metal stent was removed using an endoscopic snare, and follow-up endoscopy revealed a completely opened pyloric channel.

One month later, the LAMS was removed using a snare, and follow-up endoscopy revealed a completely opened pyloric channel (Figure 4). At the 6-month follow-up, the patient remained asymptomatic (with video).

## Technique

Endoscopic balloon dilation should ideally be performed around the 4th week; however, it is limited by the need for repeated sessions and high recurrence rates, whereas FCSEMSs are associated with a risk of stent migration. To overcome these limitations, an LAMS was selected because of its saddle-shaped design with double-walled flanges oriented perpendicular to the lumen on both sides. A stent dwell time of 4-6 weeks is recommended to prevent stent ingrowth. The application and suturing of LAMS were evaluated in a postbariatric surgery stricture, wherein stent migration was entirely prevented.^4^ In the literature, LAMSs for benign strictures demonstrated a technical success rate of 99.9%, a long-term clinical success at 72.8%, and an overall adverse event (bleeding, perforation, migration, tissue ingrowth, etc.) rate of 13.5%.^5^ Thus, LAMS placement was planned as a rescue technique in this patient with caustic-induced GOO to avoid surgery.

Long-term outcomes of LAMS use after caustic injuries remain unknown. However, according to the authors’ experience, this procedure was technically successful, and both the immediate and 6-month follow-up evaluations confirmed its safety and efficacy. The application of LAMS includes endoscopic ultrasound-guided gastroenterostomy and direct trans-stricture placement of LAMS. As demonstrated in this patients, direct trans-stricture placement of LAMS across the stenotic pyloric channel preserves the natural physiological pathway and is more straightforward and feasible when the stricture is short and can be traversed with a guidewire.^5^

A notable limitation of the current study is the relatively short follow-up period of 6 months. Further investigation through large-scale, prospective studies is needed to define the long-term clinical efficacy of LAMS and its optimal role in the management algorithm for benign GOO.

## Conclusion

In patients with caustic-induced GOO, LAMS can be used as salvage therapy to avoid surgery. Because of the paucity of evidence supporting the use of LAMS for caustic injuries, the authors recommend limiting this procedure to carefully selected patients and performing it only at highly experienced centers.

## Data Availability

The data that support the findings of this study are available on request from the corresponding author.
